# Autoantibody Biomarker Discovery in Primary Open Angle Glaucoma Using Serological Proteome Analysis (SERPA)

**DOI:** 10.3389/fimmu.2019.00381

**Published:** 2019-03-07

**Authors:** Vanessa M. Beutgen, Natarajan Perumal, Norbert Pfeiffer, Franz H. Grus

**Affiliations:** Experimental and Translational Ophthalmology, Department of Ophthalmology, University Medical Center of the Johannes Gutenberg–University Mainz, Mainz, Germany

**Keywords:** autoantibodies, biomarker, glaucoma, immunoproteomics, microarray, trabecular meshwork

## Abstract

Glaucoma is an optic neurological disorder and the leading cause of irreversible blindness worldwide, with primary open angle glaucoma (POAG) as its most prevalent form. An early diagnosis of the disease is crucial to prevent loss of vision. Mechanisms behind glaucoma pathogenesis are not completely understood, but disease related alterations in the serological autoantibody profile indicate an immunologic component. These changes in immunoreactivity may serve as potential biomarkers for glaucoma diagnostics. We aimed to identify novel disease related autoantibodies targeting antigens in the trabecular meshwork as biomarkers to support early detection of POAG. We used serological proteome analysis (SERPA) for initial autoantibody profiling in a discovery sample set. The identified autoantibodies were validated by protein microarray analysis in a larger cohort with 60 POAG patients and 45 control subjects. In this study, we discovered CALD1, PGAM1, and VDAC2 as new biomarker candidates. With the use of artificial neural networks, the panel of these candidates and the already known markers HSPD1 and VIM was able to classify subjects into POAG patients and non-glaucomatous controls with a sensitivity of 81% and a specificity of 93%. These results suggest the benefit of these potential autoantibody biomarkers for utilization in glaucoma diagnostics.

## Introduction

Glaucoma describes a group of optic neuropathies and is the leading cause of irreversible blindness. Primary open angle glaucoma (POAG) is the most common form of the disease (1), with a prevalence of 3.05% in people aged 40–80 years worldwide (2). Characteristic for glaucoma is the loss of retinal ganglion cells (RGCs) and their axons by apoptosis, leading to constant impairment of the visual field and finally complete loss of sight. Alongside old age, a major risk factor for this disorder is an elevated intraocular pressure (IOP), leading to the onset of glaucoma in 10% of affected people over the course of 5 years (3). As of today, the reduction of the IOP is the only therapy available that can prevent glaucoma progression. Cause of the IOP elevation is a deficiency in the drainage of aqueous humor (AH) from the eye via the trabecular meshwork (TM). Under normal conditions, TM cells also show fibroblast like properties, allowing them to contract and actively modulate outflow resistance. The TM in POAG is characterized by an extensive accumulation of extracellular matrix (4), the reorganization of the actin cytoskeleton (5, 6) and a decreased cellularity. This causes an overall increase of tissue rigidity leading to diminishing capability to adapt to changing IOP levels. Malfunction of the AH outflow facility finally causes an increase of IOP above physiological levels, inducing mechanical stress to the optic nerve head. The constant stress to the RGCs is thought to be one major factor in glaucoma pathogenesis, leading to apoptosis of the nervous cells (7). Albeit, molecular mechanisms behind pathological changes in the TM are not fully understood.

The subtle onset of the disease usually leads to a late diagnosis, since visual field defects are often first detectable by visual field testing when 25–35% of RGCs are already lost (8). This clearly shows the need for new diagnostic tools, capable of an early detection of reliable markers in easy accessible body fluids, like tear or serum.

Previous studies indicate an autoimmune component to the pathogenesis of glaucoma (9–11). Autoantibodies show altered levels in serum and aqueous humor of glaucoma patients. The quantity of autoantibodies against various heat shock proteins (11–14), α-fodrin (10), γ-enolase (15), vimentin (11), myelin basic protein (16), glial fibrillary acidic protein (16), and more have been found to be altered in a disease specific manner. Increased and decreased autoantibody reactivities could be detected, indicating an autoaggressive potential along with the loss of putative neuroprotective effects (17) of some autoantibodies, which could contribute to the pathogenesis of the disease.

With the goal of improving the diagnostic potential of serological biomarkers for glaucoma diagnosis, we sought to find new immunological biomarkers for POAG. To this end we used a serological proteome analysis (SERPA) approach. This technique has already been successfully applied for the identification of potential autoantibodies in other diseases, such as diabetes type 1 (18), multiple sclerosis (19), and different forms of cancer (20–22). Validation of the findings from the discovery stage SERPA are achieved by the use of the protein microarray technique, a high-throughput method for the simultaneously analysis of multiple samples (23, 24).

This work includes the discovery stage and the early validation stage of the biomarker pipeline (25) in search of reliable diagnostic biomarkers for POAG. Although several candidate biomarkers have been suggested in other studies, their diagnostic power is not sufficient yet. Based on its role in the elevation of IOP in the course of the disease, we expect to find novel autoantibodies directing target proteins in the TM.

## Materials and methods

### Sera

All samples were collected in accordance to the Declaration of Helsinki on biomedical research involving human subjects. Written informed consent was obtained from each subject to use serum samples for research purposes. The study was approved by the ethics committee of the Landesärztekammer Rheinland-Pfalz [Vote: 827.228.11 (7770)]. The subjects included in this study underwent an ophthalmic examination at the ophthalmic department of the university medical center in Mainz, Germany. For the initial screening using SERPA, six POAG samples were compared to six age and gender matched controls. In the microarray analysis, 60 sera from POAG patients and 45 sera from controls were analyzed. Inclusion criteria for POAG subjects was a diagnosis based on the European Glaucoma Society guidelines (26), according to characteristic optic nerve appearance and clinical parameters with elevated IOP, optic nerve cupping and/or characteristic visual field defects. The samples used in this study were randomly selected from available samples, only ensuring an even distribution of male and female patients using stratified random sampling with all available cases as sampling frame. Samples for the SERPA screening and for the microarray analysis were independently drawn from the same sampling pool. As control, a group of non-glaucomatous, age and gender matched subjects was used. This group comprises patients with other eye diseases and eye-healthy volunteers. Subjects with a reported autoimmune disease or other forms of glaucoma were excluded from the analysis. Other systemic diseases were not an exclusion criterion. In detail, the control group comprises 25 subjects with other eye diseases, 5 subjects with a systemic disease, 6 subjects with systemic and another eye disease, as well as 9 healthy subjects. Characteristics of the study population in the discovery and validation stage are demonstrated in Table 1. The POAG group includes patients with mild/early (MD < −6 db; 36.7%) and advanced (MD > −6 db; 63.3%) glaucomatous visual field defects, as observed by perimetry. Thereby, early POAG was regarded as a subgroup of special interest. All blood samples were allowed to clog for 30 min and were centrifuged for 10 min at 4°C and 1,000 g. The supernatant was collected and serum samples were stored at −80°C until further use.

**Table 1:** Descriptive statistics of the study population.

	**POAG**	**CTRL**
**DISCOVERY PHASE**		
Sample size	6	6
Gender (m/f)	3/3	3/3
Disease stage grading (mild/advanced)	3/3	No grading
Mean age (±SD)	66.17 ± 10.22	67 ± 11.45
**VALIDATION PHASE**		
Sample size	60	45
Gender (m/f)	30/30	23/22
Disease stage grading (mild/advanced)	22/38	No grading
Mean age (±SD)	62.75 ± 12.20	63.31 ± 15.32

### TM Tissue Procurement

Porcine eyes are a commonly used *ex vivo* model for ophthalmic research with a morphology analog to the human eye (27, 28) and therefore were used as protein source for the experiments in this study. The eye balls were collected from a local abattoir, immersed in phosphate buffered saline (PBS, Sigma) and kept on ice during transition. Eyes were processed within 5 h after enucleation. TM tissue was dissected according to Bachmann et al. (29). Briefly, eyes were opened with an equatorial cut, the posterior segment and the vitreous were discarded. Lens, iris, and ciliary body were removed with a forceps by a gentle pull. The anterior segment of the eye was washed with PBS to remove remaining iris pigment. The following steps were executed under a dissecting microscope. After removing the remnants of the pectinate ligaments, two incisions were made, flanking the TM. One was set adjacent to the line of Schwalbe, another one adjacent to the scleral spur, leading to exposure of the TM. The tissue was excised using fine scissors, avoiding contaminations with sclera and neighboring tissue. Since preparation of this tissue is a delicate task, contaminations with other tissues cannot entirely be ruled out. TM tissue of five eyes was pooled in one tube and stored in lysis buffer at −80°C until further processing.

### Protein Extraction and Precipitation

TM tissue of five eyes was disrupted, using a sonicator, in 100 μL lysis buffer [125 mM Tris-HCl, pH 7; 100 mM NaCl; 0.1% Triton-X 100; 0.1% Tween 20; 0.5% Protease Inhibitor Cocktail (Sigma)]. After 1 h incubation on ice, tissue lysates were centrifuged at 20,000 g and 4°C. Soluble proteins were collected in a new vial, the cell pellet was resuspended in fresh lysis buffer and centrifuged at 20,000 g and 4°C for additional two times. Supernatants were pooled in one tube.

Seventy-five microliters of 72% trichloroacetic acid were added to the soluble protein fraction, and incubated for 30 min on ice. Precipitated protein was centrifuged at 20,000 g and 4°C for 30 min. The supernatant was discarded and the protein pellet was washed one time with HPLC grade water and two times with Acetone. After the last washing step, the pellet was air dried and resuspended in resolubilization buffer (8M Urea; 400 mM Tris; 4% CHAPS). Total extracted protein amount was determined using a BCA Assay kit (Thermo Scientific).

### 2D PAGE

For first dimension protein separation, according to the isoelectric point, 7 cm NL pH 3–10 IPG-strips (GE Healthcare) were used. One microliter Protease Inhibitor Cocktail (Sigma), 1 μL Bromophenol blue, 1.25 μL 2M dithiothreitol (DTT) and 2.5 μL IPG buffer (pH 3–10) were added to 100 μg TM protein. Volume was adjusted to 125 μL by adding resolubilization buffer + 0.12% DeStreak Reagent (GE Healthcare). Samples were incubated at 4°C with light agitation for 30 min, applied to IPG strip holders together with the respective IPG strips. Isoelectric focusing protocols were used as previously described (30). Briefly, proteins were allowed to rehydrate for 2 h at room temperature, followed by a 12 h step at 20 V and 20°C. Afterwards proteins were focused by increasing voltage gradually over 1 h to 500 V and holding this voltage for another hour. Afterwards voltage was increased again using a gradient to 1,000 V in 0.5 h and staying at this voltage for one additional hour. This step was followed by a 0.5 h gradient to 4,000 V and 2 h a holding step at 4,000 V. The final focusing step included voltage increase to 8,000 V during a 2 h gradient followed by a 2 h holding step.

After focusing, IPG strips were equilibrated for 2 × 15 min with slight agitation in 10 mL equilibration buffer (6M Urea; 75 mM Tris; 2%SDS; 34.5% Glycerol) containing 100 mg DTT for first equilibration and 250 mg iodoacetamid for the second.

For the second dimension SDS-PAGE, 4–12% precast gradient gels were used (NuPage, Novex; Bis-Tris gels) with MES running buffer (NuPage, MES SDS running buffer, Invitrogen). The IPG strip was briefly washed in MES buffer prior to application to the gel. The strip was fixed by adding a 0.5% Agarose solution, stained with bromophenol blue. Proteins were separated at 150 V for approximate 2 h.

Gels for the creation of the fusion image spot map and preparative gels were stained using the Invitrogen colloidal blue staining kit.

### 2D Western Blot

Proteins from the 2D gels were transferred for 2 h to a nitrocellulose membrane using a wet blot system (Mini Trans-Blot Cell; Bio Rad) using a standard Towbin buffer (31). Proteins were fixed and transfer efficiency was validated by ponceau S staining (Thermo Scientific). Unspecific binding sites on the blot were blocked by incubation of the membrane in 4% non-fat dry milk in TBST (Tris buffer saline + 0.5% Tween 20) for 1 h at room temperature. After washing the membrane three times with TBST, it was incubated with patients serum (1:40 in TBST) overnight under slight agitation at 4°C. After three more washing steps, the membranes were incubated with an anti-human IgG Fc HRP secondary antibody (Goat anti-human IgG H&L (HRP); Abcam) for 1 h at room temperature with slight agitation. Following three additional washing steps with TBST, the detection of the antibody reaction was achieved by a colorimetric approach, incubating the blots with a 3,3′,5,5′-tetramethylbenzidine substrate solution (1-Step™ TMB-Blotting Substrate Solution; Thermo Fisher Scientific) for 30 s. The reaction was quenched by washing the blots in HPLC grade water.

### Imaging

Stained gels and western blots were imaged using a flatbed scanner (Epson V600). Images were scanned as 16 bit grayscale files. Spot detection, creation of the 2D fusion map and quantitative analysis was achieved by using the software Delta2D (Decodon). Spot quantities were analyzed as relative quantity of the spot, excluding background, where the total quantity of all spots on a 2D western blot is 100%. Thereby, the bias from different absolute staining intensities on individual blots is minimized.

### Total Protein Stain

After imaging, the western blot membranes were washed for 5 min in water and another 5 min in PBS. Afterwards, the membranes were incubated in PBST (PBS + 0.5% Tween 20) for 1 h. After washing three times for 2 min in water, the membranes were incubated for 2 h in colloidal gold staining solution (Colloidal Gold Total Protein Stain, Biorad). Following three further washing steps with water, the membranes were allowed to dry and scanned using a flatbed scanner system.

### LC-ESI-MS/MS

Spots of interest were cut out of three colloidal blue stained gels using a scalpel. Each protein spot was pooled with its corresponding spot from two other identical gels to allow the detection of less abundant proteins by MS. In-gel digestion was carried out according to the protocols of Shevchenko et al. (32). Gel pieces were destained, reduced and alkylated before tryptic digestion of the proteins. Prior to MS analysis, the samples were cleaned up using C18 Resin ZipTip pipette tips (Millipore), as described earlier (30, 33). The LC-system consisted of a Rheos Allegro pump (Thermo Scientific, Rockford, USA) and a PAL HTC autosampler (CTC Analytics, Zwingen, Switzerland), as described elsewhere (34). The system comprised of a 30 × 0.5 mm BioBasic C18 column (Thermo Scientific). Solvent A was LC-MS grade water with 0.1% (v/v) formic acid and solvent B was LC-MS grade acetonitrile with 0.1% (v/v) formic acid. The gradient was run for 90 min per gel spot as follows: 0–50 min, 10–35% B; 50–70 min, 35–55% B; 70–75 min, 55–90% B; 75–80 min, 90% B; 80–83 min, 90–10% B; and 83–90 min, 10% B. Continuum mass spectra data were acquired on an ESI-LTQ-Orbitrap-XL MS (Thermo Scientific, Bremen, Germany). The LTQ-Orbitrap was operated in a data-dependent mode of acquisition to automatically switch between Orbitrap-MS and LTQ-MS/MS acquisition. Survey full scan MS spectra (from *m/z* 300 to 2,000) were acquired in the Orbitrap with a resolution of 30,000 at *m/z* 400 and a target automatic gain control (AGC) setting of 1.0 × 10^6^ ions. The lock mass option was enabled in MS mode and the polydimethylcyclosiloxane (PCM) *m/z* 445.120025 ions were used for internal recalibration in real time (35). The five most intense precursor ions were sequentially isolated for fragmentation in the LTQ with a collision-induced dissociation (CID) fragmentation, the normalized collision energy (NCE) was set to 35% with activation time of 30 ms with repeat count of 10 and the dynamic exclusion segment was disabled. The resulting fragment ions were recorded in the LTQ.

The acquired continuum MS spectra were analyzed by Thermo Proteome Discoverer software (ver. 1.1.0.263; Thermo scientific). The tandem MS spectra were searched against Uniprot database (combined *Homo sapiens* and *Sus scrofa*, date:20.04.2018) using settings with peptide mass tolerance of ±50 ppm, fragment mass tolerance of ±0.5 Da and peptide charge state 1+ to 4+, using MASCOT server version 2.2.7. FDR for peptide and protein identification was set to 0.01. Carbamidomethylation of cysteine was set as a static modification, while protein oxidation of methionine were defined as dynamic modifications, enzyme: trypsin and maximum number of missed cleavages: 1. The mass spectrometry proteomics data have been deposited to the ProteomeXchange Consortium via the PRIDE partner (36) repository with the dataset identifier PXD011752.

### Microarray Fabrication

Microarrays were manufactured in our lab using a non-contact piezo-dispenser (SciFLEXARRAYER S3, Scienion, Berlin, Germany). The selected candidate antigens were purchased as recombinant human proteins (Table S1) and spotted in triplicates onto nitrocellulose coated microarray slides (AVID Oncyte, 16 Pad NC slides, Grace Biolabs, Bend, Oregon, USA). As positive and negative control spots, human IgG (Sigma), secondary antibody conjugated to alexa flour 647 and spotting buffer (PBS) were included on each array. The spotting process was carried out in a humidity chamber with humidity set to 60%. To allow optimal immobilization of the proteins on the microarray surface, slides were kept on the spotter platform to dry overnight prior to incubation.

### Microarray Incubation and Image Acquisition

Slides were incubated using incubation chambers (ProPlate Multiwell chambers, Grace Biolabs, Bend, USA), that divide the slide in 16 subarrays. The following incubation steps were performed at 4°C on an orbital shaker. To decrease background signals, the arrays were incubated with blocking buffer (Super G, Grace Biolabs, Bend, Oregon, USA) for 1 h. After removing the blocking buffer, residual buffer was washed away three times with phosphate-buffered saline containing 0.5% Tween-20 (PBST). The arrays then were incubated with 100 μL serum samples in a 1:250 dilution in PBS overnight. One subarray on each slide was incubated with PBS to serve as a negative control. Slides were washed again three times with PBST followed by incubation for 1 h at room temperature with a secondary anti-human antibody conjugated with Alexa flour 647 (Alexa Fluor® 647 AffiniPure Goat Anti-Human IgG, Fcγ fragment specific, 109-605-008, Jackson Immunoresearch) in a 1:500 dilution in PBS. After the incubation with the secondary antibody, the slides were washed two times with PBST and two times with ultrapure water. The microarray slides were dried for 2 min in a vacuum concentrator (SpeedVac, Thermo Scientific, Waltham, Massachusetts, USA).

Slides were scanned with a high resolution confocal laser scanner (428 Array Scanner, Affymetrix, Santa Clara, California, USA), yielding 16 bit TIFF images. Spot intensities were quantified with the image analysis software Imagene (Imagene 5.5, BioDiscovery Inc., Los Angeles, California, USA). Spots with poor quality were manually flagged and removed from further analysis.

### Microarray Data Pre-procession

Local background was subtracted from the median spot intensities. Negative background subtracted intensities were set to zero. Low quality spots were flagged and excluded from analysis. To correct for the unspecific binding of the secondary antibody, the signal of the negative control included on each slide was subtracted from each spot. The replicate spot intensities were averaged, yielding one mean fluorescence intensity. To correct for technical variability, the signal intensities were normalized to the IgG control spots on each subarray by median centering. To this end, the IgG median signal intensities were divided by the overall IgG signal median, yielding a normalization factor. All signal intensities on one subarray were multiplied with their correspondent normalization factor. The resulting normalized fluorescence intensities (NFI) were forwarded to statistical analysis.

### Statistical Analysis

Normality of the obtained data was assessed using one-sample Kolmogorov–Smirnov (K-S) test. Data obtained by SERPA was normally distributed (K-S test not significant at alpha = 0.05), therefore, differences in spot quantities in the SERPA discovery phase were assessed using student's *t*-test. Some variables in the microarray data did not follow a normal distribution (K-S *p* < 0.05) and consequently changes in autoantibody levels were assessed using Mann–Whitney *U*-test. A *p*-value of <0.05 was considered statistical significant. In order to obtain a more robust statistic and reduce the influence of outliers in the microarray data set, values below the 5th and above the 95th percentile in each group were excluded from analysis. Kruskal–Wallis H test was used for multiple group comparisons. ROC curves were computed using artificial neural networks (ANN). The multilayer perceptron (MLP) networks were trained by randomly using 70% of the cases as training set and 30% as test set. ROC curves and AUROCC values refer to test performance only on the test set. Missing data in the microarray data set was replaced by the respective group mean for the ANN model building. The statistical data analysis was performed using Statistica (Statistica 13, Statsoft, Tulsa, Oklahoma, USA).

## Results

### Discovery of Candidate Autoantigens by SERPA

**Figure 1:**
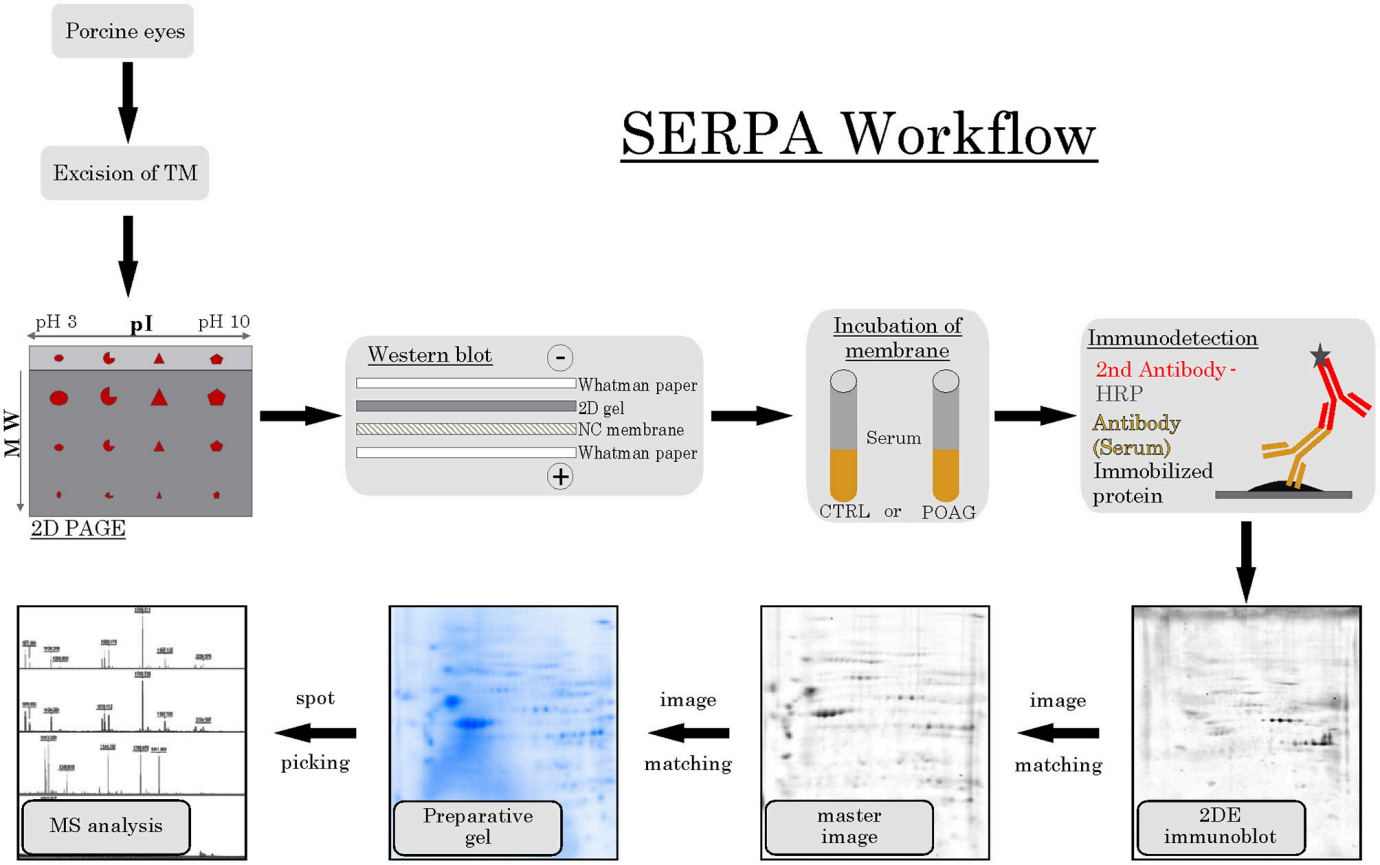
SERPA workflow. TM tissue was excised from porcine eyes and analyzed by 2DE PAGE, separating the proteins according to their isoelectric point (pI) and their molecular weight (MW). Next, proteins were transferred to a nitrocellulose (NC) membrane using a wet blot system. Then, the membranes containing the immobilized protein were incubated with either CTRL or POAG serum, using the contained autoantibodies as primary antibodies. As secondary detection antibody, anti-human antibodies conjugated to horse radish peroxidase (HRP) were used and visualized by a colorimetric detection. The resulting 2DE immunoblots were matched to their corresponding spots on a colloidal blue stained master image, computed as average pattern from three independent 2DE gels. Spots of interest were then identified on preparative gels and prepared for MS analysis.

**Figure 2:**
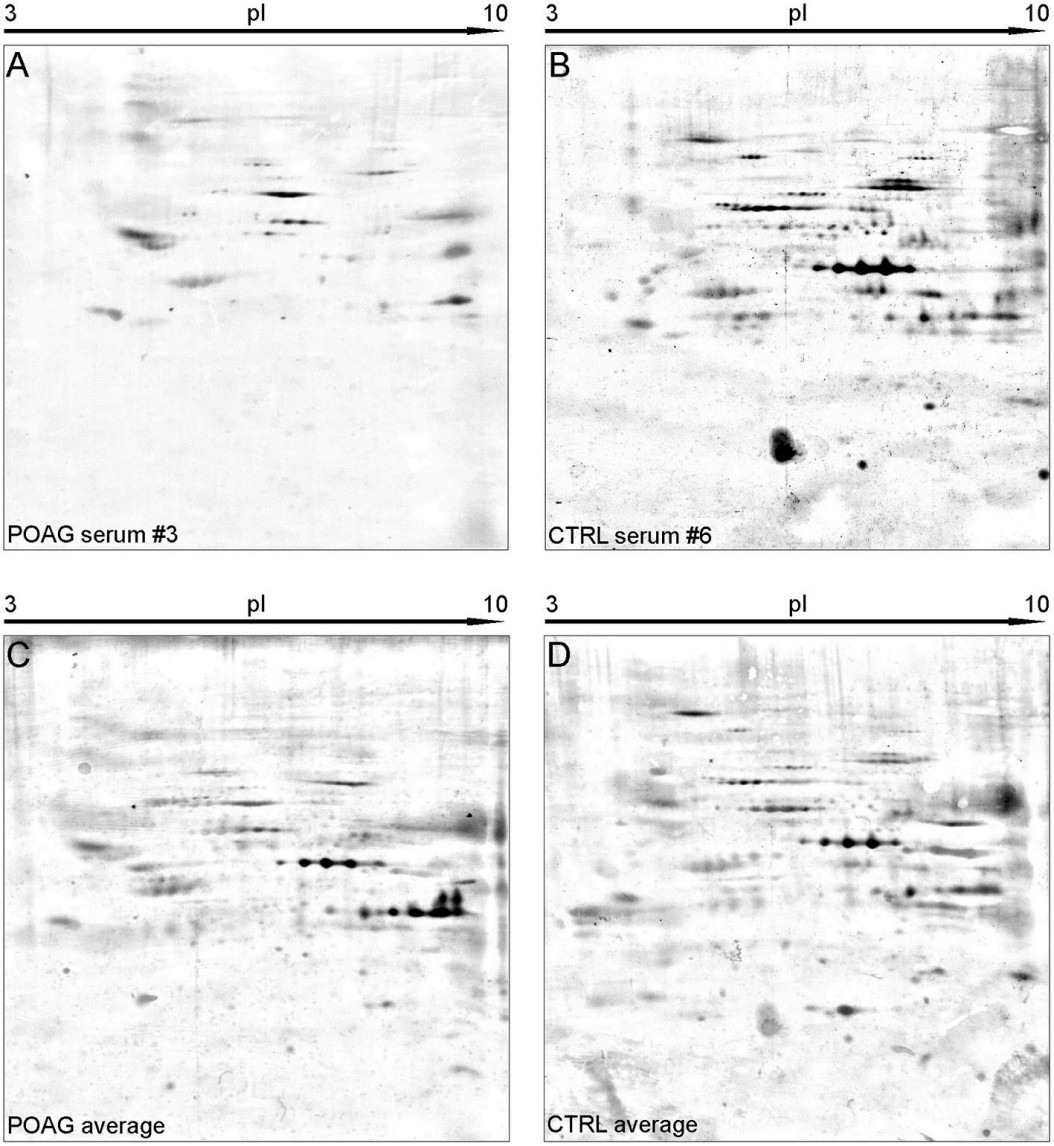
Representative SERPA immunoblots. **(A,B)** Show 2DE immunoblots of individual sera form either POAG **(A)** or CTRL group **(B)**. **(C,D)** Show the average pattern for the POAG and the CTRL group computed from all immunoblots by Delta2D. A high abundance of diverse autoantibodies could be detected in both groups. Interesting spots were selected upon their different gray intensity between the two groups and forwarded to MS analysis.

**Figure 3:**
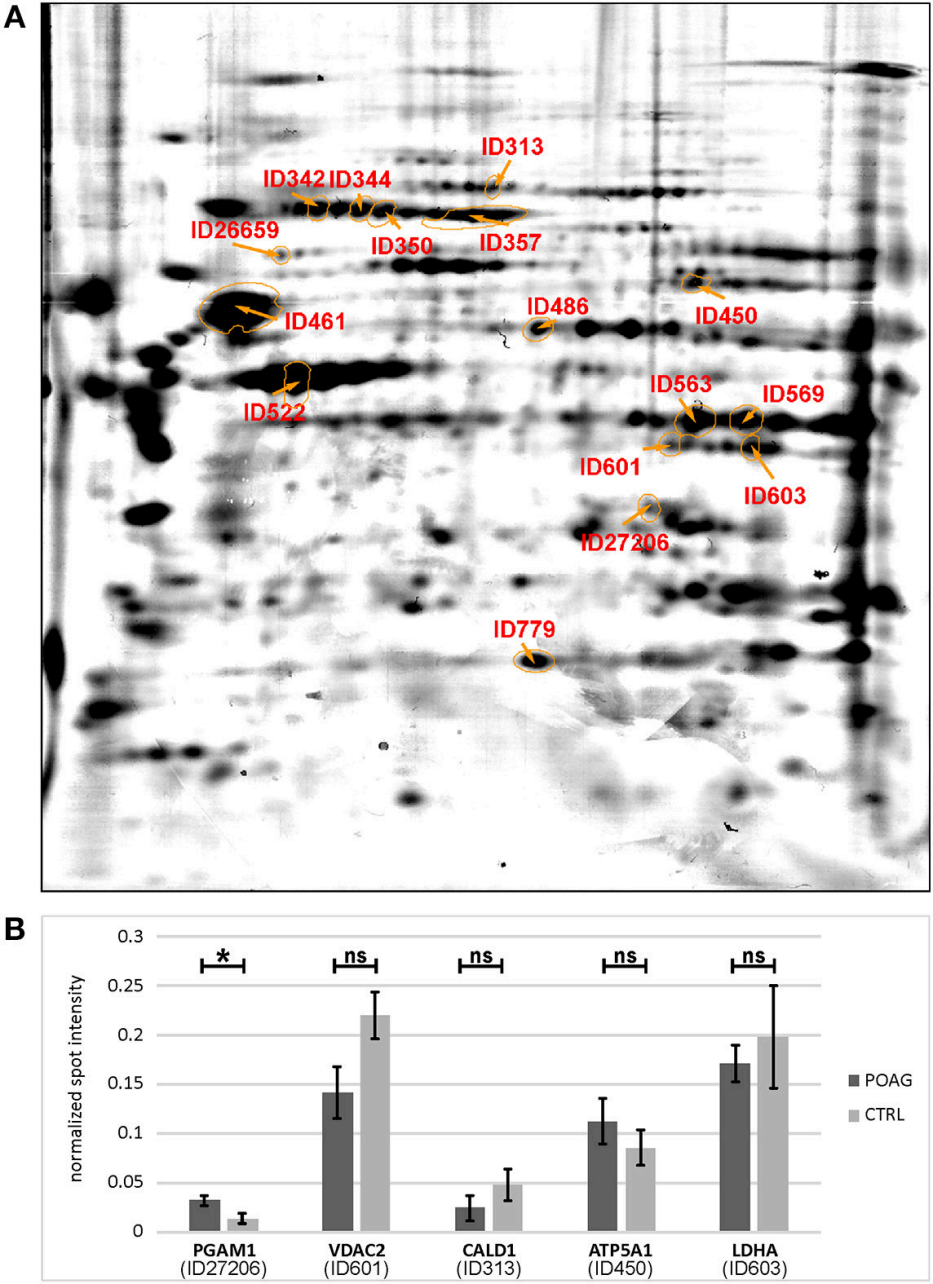
Quantification of spot intensities and protein identification. **(A)** The picture shows a spot map computed of three individual colloidal blue stained 2DE gels, reflecting an average image of the porcine TM proteome. Spots of interest were labeled with IDs and identified by MS analysis (Table 2). **(B)** Comparison of immunoblot spot intensities between groups. Normalized spot intensities are shown for PGAM1, VDAC2, CALD1, ATP5A1, and LDHA. Asterisk indicates significance at *p* < 0.05, ns, not significant.

The aim of this analysis was the identification of potential autoantibodies to antigens in the TM. In this discovery phase study, serum of six POAG patients and six age and gender matched controls (CTRL) were analyzed for autoreactive antibodies against proteins derived from porcine TM tissue lysates. To this end, we used SERPA as a widely used immunoproteomic method for the identification of serological autoantibodies (Figure 1). TM tissue lysates were separated by 2DE and transferred onto nitrocellulose membranes. Membranes were subsequently incubated with individual serum samples from the POAG or the control group. As shown in Figure 2, a broad range of antigen-autoantibody-complexes were detected in both groups, but autoantibody profiles also strongly varied amongst individuals. To find differences in the autoantibody repertoire of the two groups, protein spots on the immunoblots were analyzed and spot intensities were quantified using Delta2D (Decodon). Raw spot quantities were normalized to the equalized total signal intensities of the immunoblots to prevent bias from blot incubation. A spot map was created of three colloidal blue stained 2DE gels using TM tissue lysates. In Delta2D images of these gels were computed to obtain a single master image showing the average spot pattern of the three gels (Figure 3A). Spots that showed differences between groups in the quantity of their average gray value (Figure 3B) and could be visually identified on colloidal blue stained gels were selected for identification by MS. The spots of interest from the 2DE western blots were matched with the correspondent spot on the spot map. To help the identification of the spots on the spot map, a total protein stain using colloidal gold was applied to the western blot membranes. This allowed for the application of the image warping option in Delta2D, were spot pattern of two different images can be aligned. This enabled a reliable identification of the immunoreactive spots on the preparative gels used for the MS analysis. The spots of interest were cut out in three preparative gels and pooled to increase the protein amount for MS analysis. Spots were identified by MS analysis as shown in Table 2.

**Table 2:** Identified 2DE spots by MS analysis.

**Spot ID**	**Gene name**	**Protein**	**Accession**	**Sequence coverage (%)**	**# Peptides**	**# AAs**	**MW [kDa]**	**calc. pI**	**Score**	**Total peptide intensity/spot (%)**
ID313	CALD1	Caldesmon	Q05682	1.51	3	793	93.2	5.66	124.08	100.00
ID342	HSPA7	Putative heat shock 70 kDa protein 7	P48741	3.54	8	367	40.2	7.87	348.76	100.00
ID344	HSPA2	Heat shock-related 70 kDa protein 2	P54652	23.63	147	639	70.0	5.74	6070.68	26.78
ID350	HSPA1B	Heat shock 70 kDa protein 1B	Q6S4N2	28.71	169	641	70.1	5.82	7349.82	32.62
ID357	ALB	Serum albumin	P08835	44.15	287	607	69.6	6.49	13530.87	99.25
ID450	ATP5A1	ATP synthase subunit alpha, mitochondrial	P80021	20.25	83	553	59.7	9.19	3511.75	85.46
ID461	VIM	Vimentin	P02543	52.15	412	466	53.6	5.12	18264.69	66.19
ID486	ENO1	Alpha-enolase	P06733	20.74	99	434	47.1	7.39	5076.76	68.38
ID522	ACTB	Actin, cytoplasmic 1	P60712	33.33	243	375	41.7	5.48	12218.77	50.79
ID563	ANXA2	Annexin A2	P19620	56.64	380	339	38.5	6.93	15255.93	52.92
ID569	ANXA2	Annexin A2	P19620	53.98	248	339	38.5	6.93	10170.05	70.66
ID601	VDAC2	Voltage-dependent anion-selective channel protein 2	P45880	24.83	69	294	31.5	7.56	3231.96	70.09
ID603	LDHA	L-lactate dehydrogenase A chain	P00339	32.83	86	332	36.6	8.07	2434.55	45.75
ID779	SOD1	Superoxide dismutase [Cu-Zn]	P04178	29.41	46	153	15.9	6.52	1699.91	94.41
ID26659	HSPD1	60 kDa heat shock protein, mitochondrial	P10809	24.43	118	573	61.0	5.87	5271.25	95.14
ID27206	PGAM1	Phosphoglycerate mutase 1	P18669	35.43	100	254	28.8	7.18	4999.49	80.05

A total of 16 autoantigens could be identified by LC-ESI-MS/MS. They all showed variations in mean autoantibody levels between groups, with in- and decreased signal intensities, but only one spot showed significant changes (Table 3). The spot identified as phosphoglycerate mutase 1 (PGAM1) showed significant differences between groups (*p* = 0.03), as analyzed by student's *t*-test. Other autoantigens that have not yet been described in the context of glaucoma were identified as ATP synthase subunit alpha (ATP5A1), Caldesmon (CALD1), Voltage-dependent anion-selective channel protein 2 (VDAC2), and L-lactate dehydrogenase A (LDHA) but failed to show significant group differences. Furthermore, some previous identified glaucoma autoantigens including heat shock protein 60 dDA (HSPD1) (12, 13), heat shock protein 70 kDa (HSPA1B) (11), vimentin (VIM) (11), α-enolase (ENO1) (37) and superoxide dismutase 1 (SOD1) (38) could be detected (Table 2). Considering the caveats of the colorimetric detection used in this approach and the low statistical power of this discovery screening approach, it has been decided to include all detected autoantigens in the microarray validation step to analyze the respective autoantibody levels in a method providing a higher dynamic range and capable to process a larger number of samples. Thus, this enables the detection of smaller effects that might be missed in the initial discovery.

**Table 3:** Quantification of normalized signal intensities from SERPA immunoblots and statistical significance (*p*-value *t*-test).

	**POAG mean**	**CTRL mean**	**POAG *SD***	**CTRL *SD***	***t*-value**	***p*-value**
PGAM1	0.03	0.01	0.01	0.01	2.50	0.03
VDAC2	0.14	0.22	0.07	0.06	−2.20	0.05
CALD1	0.02	0.05	0.03	0.04	−1.16	0.27
ATP5A1	0.11	0.09	0.06	0.04	0.92	0.38
HSPA7	0.33	0.20	0.33	0.13	0.87	0.40
ANXA2 (ID563)	0.61	1.01	0.21	1.11	−0.85	0.42
ENO1	0.17	0.38	0.08	0.60	−0.84	0.42
HSPD1	0.09	0.06	0.08	0.03	0.79	0.45
SOD1	0.23	0.38	0.14	0.62	−0.59	0.57
ALB	0.81	0.55	1.28	0.35	0.49	0.63
LDHA	0.17	0.20	0.04	0.13	−0.48	0.64
ANXA2 (ID569)	0.53	0.42	0.61	0.34	0.39	0.71
HSPA2	0.18	0.21	0.07	0.17	−0.30	0.77
VIM	1.27	1.09	1.29	0.74	0.28	0.78
ACTB	0.44	0.39	0.29	0.30	0.27	0.80

*Mean values and standard deviation (SD)*.

### Protein Microarray Analysis for the Validation of Putative Autoantibodies

**Figure 4:**
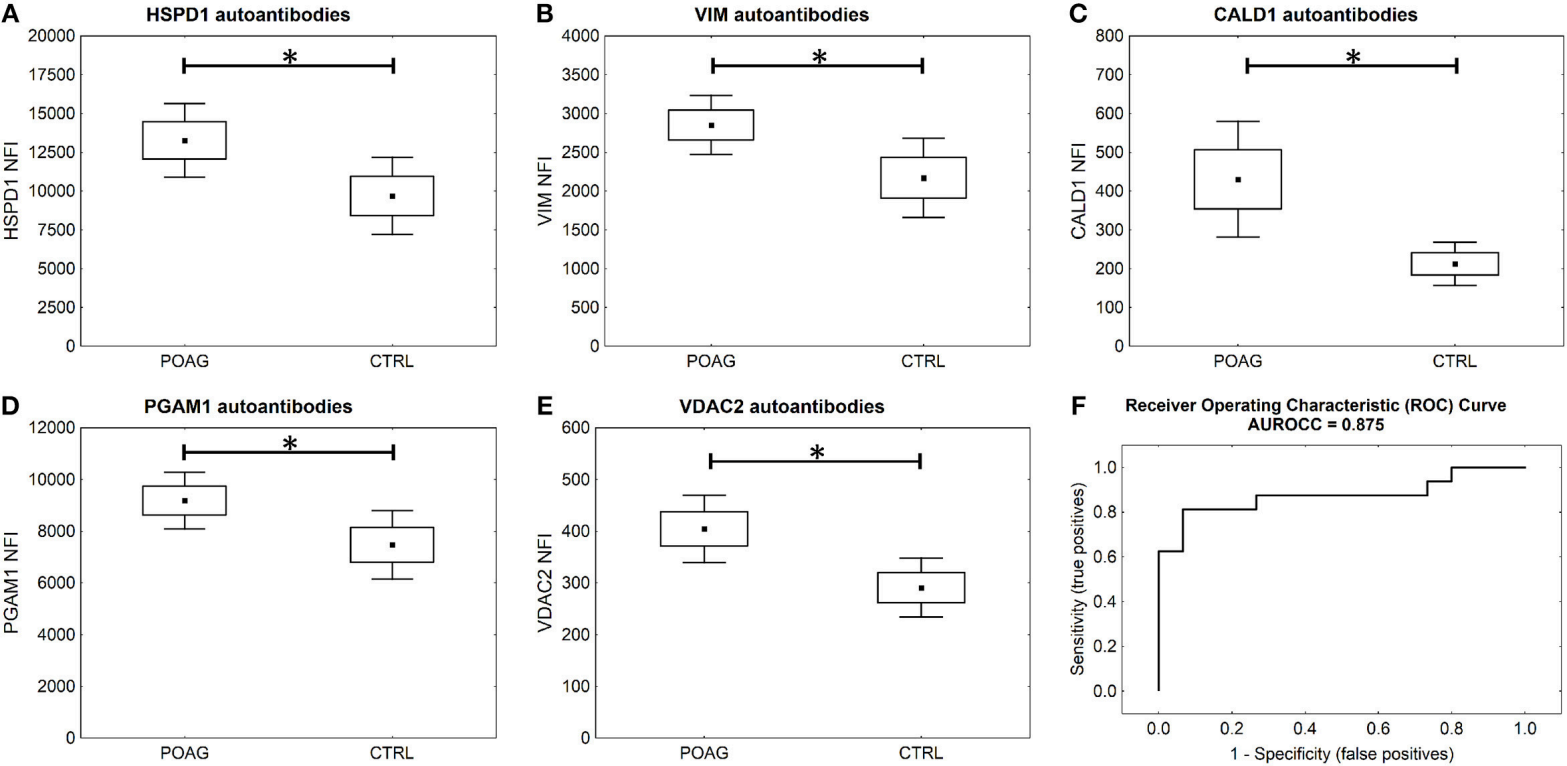
Microarray results and ROC curve. **(A–E)** Microarray analysis shows significant higher serological levels of HSPD1, VIM, CALD1, PGAM1, and VDAC2 antibodies in POAG (*U* test *p* < 0.05). Small squares indicate mean fluorescent intensities, boxes present the standard error of the mean (SE), whiskers show 1.96 times SE. ^*^$\hat{=}$ *p* < 0.05; ns $\hat{=}$ “not significant” **(F)** ROC curve for five autoantibody-marker panel by use of ANNs.

To analyze the autoantibody distribution of the detected candidates in a larger sample size, customized microarray slides were fabricated using commercially available recombinant proteins (Table S1). This approach yields fluorescence intensities for each tested autoantigen that correlate with the serological autoantibody level. Autoantibody levels of the selected candidates were analyzed in serum samples of 60 POAG patients and compared to an age and gender matched control group of 45 individuals. The received data was pre-processed and normalized to IgG control spots on each subarray. Descriptive statistics of the obtained data are shown in Table 4. Kolmogorov-Smirnov one-sample test for normality indicated, that the data is not normally distributed. Therefore, non-parametric Mann-Whitney *U* test was applied to analyze the data for alternating autoantibody distributions in POAG patients and the control group. This analysis revealed a significant increase of autoantibody levels against VIM, HSPD1, PGAM1, VDAC2, and CALD1 (*p* < 0.05, Figures 4A–E) in POAG compared to the control group. To investigate, whether the presence of other eye or systemic diseases has an influence on the tested autoantibody levels, we further analyzed the control group. To this end we divided the control group into four subgroups with regard to other diseases, comprising subjects with “other eye disease” (*n* = 25), “systemic disease” (*n* = 5), “systemic + other eye disease” (*n* = 6) and “none” (*n* = 9). Kruskal–Wallis *H*-test revealed no significant (*p* ≥ 0.05) difference among these subgroups.

**Table 4:** Normalized fluorescence intensities (NFI) obtained by protein microarray analysis.

	**CTRL mean**	**POAG mean**	**CTRL SE**	**POAG SE**	**CTRL valid *N***	**POAG valid *N***	***p*-value**
CALD1	212.16	430.47	28.54	76.10	35	45	0.023
HSPD1	9684.98	13267.04	1263.34	1210.67	41	55	0.015
ATP5A1	233.12	271.94	67.09	126.36	12	21	0.385
VIM	2171.83	2853.60	260.29	193.24	41	57	0.014
ENO1	353.22	393.12	70.63	71.74	22	33	0.858
VDAC2	291.00	404.68	29.06	33.37	36	55	0.038
LDHA+B	91.98	106.99	17.09	25.53	17	25	0.839
SOD1	280.79	340.55	38.76	37.20	38	52	0.299
ANXA2	5229.18	5594.91	499.77	337.74	30	41	0.200
PGAM1	7475.67	9186.97	676.15	558.01	39	52	0.018

*Mann**–**Whitney U***-***test for evaluation of group differences. Mean values and standard error (SE)*.

To evaluate the potential of the identified markers to discriminate between POAG and non-glaucomatous subjects we used ANNs. The MLP network was trained using a training set consisting of 70% randomly selected samples from the microarray data set of these five markers. To assess the classification power, the network was deployed on the remaining 30% of the data, the test set. Specifications of the ANN can be found in Table S2.The trained network was able to classify the POAG patients from the control group with a sensitivity of 81% and a specificity of 93% (Figure 4F). With an area under the roc curve (AUROCC) of 0.875 the test yields a good accuracy.

**Figure 5:**
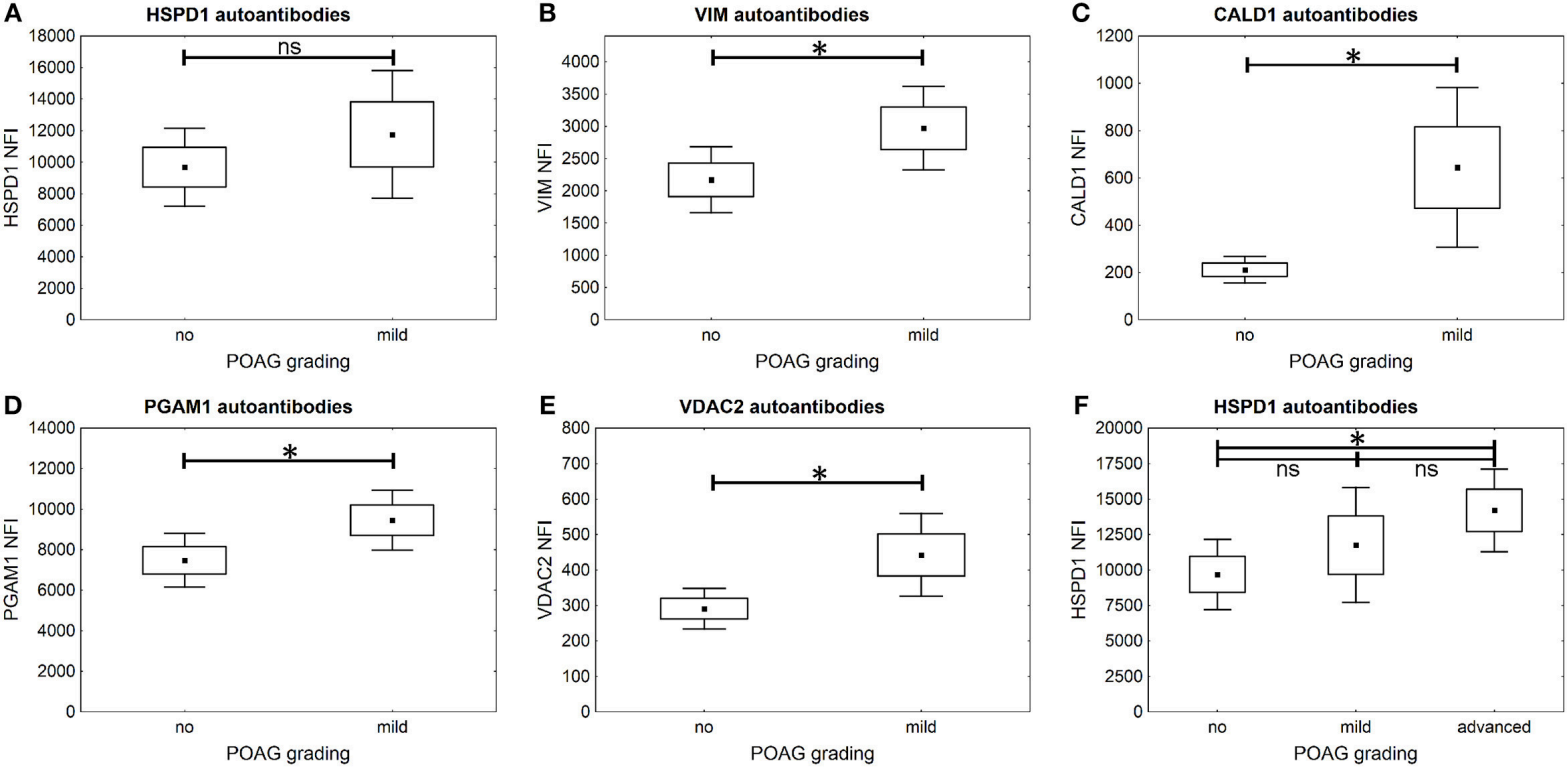
Comparison of autoantibody levels in mild POAG and CTRL (no). Small squares indicate mean fluorescent intensities, boxes present the standard error of the mean (SE), whiskers show 1.96 times SE. ^*^$\hat{=}$ *p* < 0.05; ns$\hat{=}$ “not significant” **(A–E)** The microarray analysis shows that autoantibodies to VIM **(B)**, CALD1 **(C)**, PGAM1 **(D)**, and VDAC2 **(E)** already show significantly higher levels in early POAG (*p* < 0.05), but not autoantibodies to HSPD1 **(A)** (*p* = 0.38). **(F)** Kruskal–Wallis *H*-test shows significant differences in HSPD1 autoantibody levels between different POAG gradations. The mean HSPD1 autoantibody levels increase with glaucoma progression and show significant differences between CTRL (no) and advanced POAG (*p* = 0.013).

It is further of special interest to enhance glaucoma diagnostics at an early stage of disease progression, when glaucomatous visual field damage is still minor. Therefore, a subgroup of POAG subjects, being at an early disease stage with mild visual field defects, was analyzed separately. The autoantibody levels of PGAM1, VDAC2, CALD1, and VIM are significantly higher in the subgroup of patients with mild POAG (*p* < 0.05) (Figures 5B–E). HSPD1 autoantibody levels showed no significant difference in mild POAG (Figure 5A). Kruskal-Wallis H test however, revealed a significant change in HSPD1 autoantibodies between different POAG gradations (*p* = 0.017). The mean level increases with visual field damage progression (Figure 5F).

## Discussion

Glaucoma diagnostic as it is today is time and cost intensive. Only experienced ophthalmic medical staff can evaluate glaucomatous damages of the optic nerve and even then, the diagnosis often is a matter of subjective interpretation. Clinical signs of the damage regularly only show, when the disease is far in progression. In most cases, 25–35% of the retinal ganglion cells underwent apoptosis before glaucoma is first diagnosed. To help the medical staff with the assessment of glaucoma and to provide a fast and objective test that can be included in routine examinations, reliable biomarkers need to be established in easy monitorable body fluids, such as tear or serum. Regarding autoantibody levels as potential biomarkers fulfilling these requirements, there is the caveat that no single marker is specific enough to classify healthy subjects from diseased. As already shown in cancer diagnostics (39–41), a set of markers needs to be established to enable sufficient discrimination, since fluctuations of autoantibody levels can occur in higher magnitude, even in healthy individuals. In an earlier study, the data of nine autoantibody biomarkers in serum and aqueous humor samples was used to train an ANN. The created network was able to classify the subjects in the test set with an AUROCC of 0.93 (38). But the translation of this biomarkers to an actual diagnostic test to be used in clinical routine is no trivial task, since multiplexing analysis of autoantibody concentration in a point-of-care device (POCD) with up to nine analytes has not been established yet. The challenge now is to find a set of biomarkers that is small enough to be deployed in a POCD but robust enough to allow testing with high specificity and sensitivity.

In this study, our goal was to identify novel glaucoma specific biomarkers by SERPA. To this end, we analyzed sera of six POAG patients and six non-glaucomatous individuals. With this approach, we were able to identify 16 autoantigens, but only PGAM1 autoantibodies showed significant elevated levels in POAG. To validate the observations made in this discovery phase, the identified candidates were subjected to a protein microarray analysis. Sera of 60 POAG patients and 45 controls were tested for their autoantibody levels against these target antigens. Here, VDAC2, CALD1, and PGAM1 autoantibodies have shown to be elevated in serum of POAG patients and also in the subgroup of POAG patients with an early disease stage. Therefore, they might be used as novel serological biomarkers that improve early glaucoma diagnosis.

VDAC2 is a voltage-dependent anion channel in the outer mitochondrial membrane. It is a ~30 kDa transmembrane protein (42) and is important for the translocation of small molecules across the membrane. The porin does not only act as channel for ATP and ions but can also release the apoptosis inducing cytochrome c by interacting with proteins of the Bcl-2 family (43, 44). Although, the mechanisms behind RGC death in glaucoma are not yet understood entirely, it is suggested, that the vast majority of RGCs undergo apoptosis, thereby underlining a potential role for this mitochondrial porin in the pathogenesis. Moreover, apoptosis triggered by various mechanisms can also be observed in the glaucomatous TM (45). VDAC2 was also found to be upregulated in glaucomatous rat RGCs (46), strongly demonstrating the role of apoptosis in RGC death. The deregulation of VDAC2 is a possible trigger for the onset of autoimmunity, hence the elevation of VDAC2 antibodies might be the result of the overexpression of this protein, as well as apoptotic events (47, 48). Antibodies to VDAC can also be found in sera of autistic children and have shown to induce apoptosis in human neuroblastoma cells (49), suggesting that the presence of these autoantibodies could not only be a mere expression of the pathological changes, but also enforces their progression in a synergetic manner.

Caldesmon, the gene product of CALD1, acts as a modulator of the actomyosin network by interacting with actin and cadherin, involved in cell-cell and cell-matrix adhesions. It regulates the cell contractility in a Ca^2+^–dependent manner. CALD1 overexpression in TM cells leads to actin cytoskeleton reorganization and in high abundance to disruption of adherens junctions (50). These molecular changes cause a relaxation of the tissue and thereby an increase of TM outflow facility. Therefore, CALD1 has been proposed for gene therapy of glaucoma (50, 51). Reorganization of the actin cytoskeleton has been observed in glaucomatous TM (5, 6) leading to increased tissue rigidity and failure to actively regulate IOP. Thus, we hypothesize that a participation of CALD1 deregulation in these processes cannot be ruled out. Pathologic changes in CALD1 expression, incomplete protein folding or post-translational modification could induce autoimmunity to this antigen. However, the effects and the origin of the identified CALD1 autoantibodies remain elusive and need to be addressed in further studies.

PGAM1 encodes the protein phosphoglycerate mutase 1 and is involved in the glycolytic pathway. Here it acts as an enzyme catalyzing the reaction from 3-phosphoglycerate to 2-phosphoglycerate (52). Autoantibodies to PGAM1 have been found increased in multiple sclerosis and neuromyelitis optica and have been suggested as marker for neuroinflammatory diseases (53, 54). Inflammatory processes are also prevalent in glaucoma as shown in experimental glaucoma models and in clinical studies, as reviewed by Russo et al. (55) and Soto et al. (56). This leads to the hypothesis, that PGAM1 autoimmunity can be triggered by neuroinflammatory conditions also appearing in glaucoma pathogenesis.

Levels of HSPD1 and VIM antibodies have already been described to be altered in glaucomatous diseases. Antibodies against the 60 kDa heat shock protein, encoded by HSPD1, were amongst the first to be detected in connection with glaucoma (12, 13) and are consistently increased throughout different study populations (57). The results obtained in the present study further confirm these findings. The increased VIM antibody levels detected in the present study however, are inconsistent with an earlier study from 2008 (16), in which antibody levels against optic nerve antigens were found to be downregulated in POAG and normal tension glaucoma using western blot analysis.

Overall, training of ANNs using a 5-biomarker-panel of the three new biomarker candidates with HSPD1 and VIM yielded a test that was able to classify POAG from CTRL with 81% sensitivity at 93% specificity and an AUROCC of 0.875.

The findings of this study are a further step to the development of a glaucoma rapid test that will identify early stage POAG showing only minor clinical signs. This would allow diagnostic testing not only for trained glaucoma specialists and would therefore be suitable for general health examinations. This can promote an early detection of the disease and allow an earlier start of treatment, thereby preventing glaucoma progression and preserve patients from severe vision loss. Autoantibodies as serological markers are accessible using minimal invasive methods and therefor provide a promising approach to enhance diagnostics.

Although the alteration of autoantibody levels to several ocular antigens in glaucoma has also been shown in different other studies (9–16, 58), it remains elusive, whether they are causative for pathological changes or just a byproduct that arises from them. Some studies provide evidence that endogenous antibodies can enter living cells and even promote or protect from apoptosis (59–61). Also, accumulation of IgG in the TM has been shown (62) and antibody levels in the aqueous humor are widely consistent with serum levels (38). The specific effects of autoantibodies to this tissue however, have not yet been analyzed. The hypothesis of the detected autoantibodies being an epiphenomenon seems to be more obvious. The TM in POAG suffers from a loss in cellularity (63) but the mechanisms of cell loss have not been clarified conclusively. Besides autophagy and necrosis, apoptosis has been discussed to induce cell death in glaucomatous TM (64, 65). An increased number of apoptotic and (postapoptotic) necrotic events can be a trigger for the emergence of autoantibodies to intracellular proteins (48). The cleavage of cellular components during apoptosis can even lead to the generation of neoepitopes (66). Furthermore, disease related post-translational modifications of the proteins can be an additional trigger for the occurrence of highly specific autoantibodies (67). In this case, the autoantibodies are an epiphenomenon of the damage of the tissue and/or induced by related modifications of the antigen. Both possibilities are supported by multiple circumstantial evidences and the concluding proof, of which mechanism is applicable in POAG remains a task for future studies. But regardless of their origin or effect, autoantibodies may serve as valuable disease biomarkers, as has also been demonstrated in other neurodegenerative diseases as Parkinson's disease (68) or Alzheimer's disease (69, 70). However, an important task for future evaluation studies to improve the reliability of the identified biomarkers is their investigation in other chronic and acute diseases. Also, it needs to be defined if they have a role in the natural autoantibody repertoire to gain further insights into their function in health and disease.

## Conclusion

With VDAC2, PGAM1, and CALD1, new autoantibodies have been identified in association with POAG by the application of immunoproteomic methods. A combination of these and former known autoantibodies have shown to be suitable biomarker candidates for the diagnosis of POAG, even in early disease stages. The application of advanced bioinformatic analysis using artificial neural networks improves the value of these biomarkers by building models based on a panel of five candidate biomarkers. Applied in a rapid test, available not only to glaucoma specialists, they could help to promote early POAG detection and reduce the probability of severe vision loss by timely onset of treatment. To this end however, the evaluation of test results in larger, more heterogeneous study populations is necessary. Likewise, the search for even better biomarkers and biomarker combinations needs to continue, to enable the development of reliable rapid test immunoassays as a new standard tool in glaucoma diagnostics.

## Data Availability

The MS dataset is publicly available at the PRIDE database (https://www.ebi.ac.uk/pride/archive/) with the identifier PXD011752.

## Author Contributions

VB planned the experimental design of this study, performed SERPA and microarray analysis, interpreted the data, and wrote the manuscript. NaP performed mass spectrometric analysis. FG and NaP critically revised the manuscript. FG and NoP contributed to conception of the study and provided resources.

### Conflict of Interest Statement

The authors declare that the research was conducted in the absence of any commercial or financial relationships that could be construed as a potential conflict of interest.

## Abbreviations

ANN: artificial neural network
AUROCC: area under ROC curve
IOP: intraocular pressure
MS: mass spectrometry
MLP: multilayer perceptron
POAG: primary open angle glaucoma
POCD: point-of-care device
RGC: retinal ganglion cells
ROC: receiver operator characteristic
SERPA: serological proteome analysis
TM: trabecular meshwork.
