# Supplementary material for: Autoantibody Biomarker Discovery in Primary Open Angle Glaucoma Using Serological Proteome Analysis (SERPA)
Source: Front Immunol. 2019 Mar 7;10:381. doi: 10.3389/fimmu.2019.00381 (PMC6417464; doi:10.3389/fimmu.2019.00381)
Supplement: Supplementary file 1 [file Table_1.docx]

Supplementary Material

Autoantibody biomarker discovery in primary open angle glaucoma using serological proteome analysis (SERPA)

Vanessa M. Beutgen, Natarajan Perumal, Norbert Pfeiffer, Franz H. Grus*

Experimental and Translational Ophthalmology, Department of Ophthalmology, University Medical Center of the Johannes Gutenberg – University Mainz, Mainz, Germany

*Corresponding author:

Franz H. Grus

Dept. of Experimental Ophthalmology

University Medical Center

Johannes Gutenberg University Mainz

Langenbeckstr. 1

55131 Mainz, Germany

Email: fgrus@eye-research.org

Phone: +49(0)6131-17-3328 / Fax: +49-(0)6131-4970563

Table S1 Antigens used for microarray analysis.

| Gene | Protein name | Company |
| --- | --- | --- |
| CALD1 | Caldesmon | Abcam |
| HSPD1 | 60 kDa heat shock protein, mitochondrial | Enzo life science |
| ATP5A1 | ATP synthase subunit alpha, mitochondrial | Aviva systems biology |
| VIM | Vimentin | Progen |
| ENO1 | Alpha-enolase | Abcam |
| VDAC2 | Voltage-dependent anion-selective channel protein 2 | Abcam |
| LDHA/LDHB | L-lactate dehydrogenase A / B chain | Abcam |
| SOD1 | Superoxide dismutase [Cu-Zn] | Sigma |
| ANXA2 | Annexin A2 | Abcam |
| PGAM1 | Phosphoglycerate mutase 1 | Aviva systems biology |

Table S2 Artificial neural network.

| Net. name | Training perf. | Test perf. | Training algorithm | Error function | Hidden activation | Output activation |
| --- | --- | --- | --- | --- | --- | --- |
| MLP 5-11-2 | 71.23288 | 83.87097 | BFGS 13 | Entropy | Tanh | Softmax |
